# A Novel Process for Cadaverine Bio-Production Using a Consortium of Two Engineered *Escherichia coli*

**DOI:** 10.3389/fmicb.2018.01312

**Published:** 2018-06-19

**Authors:** Jing Wang, Xiaolu Lu, Hanxiao Ying, Weichao Ma, Sheng Xu, Xin Wang, Kequan Chen, Pingkai Ouyang

**Affiliations:** ^1^State Key Laboratory of Materials-Oriented Chemical Engineering, College of Biotechnology and Pharmaceutical Engineering, Nanjing Tech University, Nanjing, China; ^2^College of Bioengineering and Biotechnology, Tianshui Normal University, Tianshui, China

**Keywords:** cadaverine, bio-based polyamides, microbial consortia, L-lysine, renewable sources, CRISPR-Cas9, feeding strategy

## Abstract

Bio-production of cadaverine from cheap carbon sources for synthesizing bio-based polyamides is becoming more common. Here, a novel fermentation process for cadaverine bio-production from glucose was implemented by using a microbial consortium of two engineered *Escherichia coli* strains to relieve the toxic effect of cadaverine on fermentation efficiency. To achieve controllable growth of strains in the microbial consortium, two engineered *E. coli* strains grown separately on different carbon sources were first constructed. The strains were, an L-lysine-producing *E. coli* NT1004 with glucose as carbon source, and a cadaverine-producing *E. coli* CAD03 with glucose metabolism deficiency generated by modifying the PTS^Glc^ system with CRISPR-Cas9 technology and inactivating cadaverine degradation pathways. Co-culturing these two engineered *E. coli* strains with a mixture of glucose and glycerol led to successful production of cadaverine. After optimizing cultivation conditions, a cadaverine titer of 28.5 g/L was achieved with a multi-stage constant-speed feeding strategy.

## Introduction

Polyamides (PA), commonly known as nylon, are widely used in numerous industries and the demand for PA products further increases with the development of industry (Tabor and Tabor, 1985). Increasing exhaustion of fossil fuels (Kim et al., 2015b) and strengthening consciousness of environmental protection are the major driving factors for increased development of environmentally friendly materials from biological sources (Mekonnen et al., 2016). Among several types of biomass derived from polymers, bio-based polyamides have been the choice for an alternative to traditional petroleum-based polyamides (Kind and Wittmann, 2011; Schneider and Wendisch, 2011; Bhatia et al., 2016). In contrast to the even-carbon diamines derived from petroleum, one of the important blocks for bio-based polyamides is cadaverine, a five-carbon diamine (Kind et al., 2014). Due to the specific properties of odd-carbon, polyamides based on cadaverine exhibit excellent and well-known material properties and could compete with the conventional petroleum-based polyamides in all examined fields (Eltahir et al., 2014; Ma et al., 2015a).

Environmentally friendly and efficient bio-production of cadaverine is the most important process for bio-based PA5.X manufacturing (Kind et al., 2014; Kim et al., 2015c). Considerable efforts in biosynthesis of cadaverine have been made in the last two decades (Takatsuka et al., 2000). A common method is whole-cell bioconversion where cadaverine is produced from the natural amino acid lysine through overexpression of the *Escherichia coli* lysine decarboxylase CadA (Mimitsuka et al., 2007) or LdcC (Kind et al., 2010). A high rate of conversion of about 80% lysine to cadaverine was successfully achieved by Kim et al. (2015a) using whole cells at a high concentration of 1.75 M lysine. Their results also identified that pyridoxal-5′-phosphate (PLP) as a critical control factor for the biotransformation of lysine to cadaverine (Kim et al., 2015a). Furthermore, cadaverine production using immobilized cells has also been reported recently. Barium alginate was selected as a matrix for immobilization (Bhatia et al., 2015) and 123 h of continuous cadaverine production was performed in a 14 mL reactor resulting in 466.5 g of cadaverine (Kim et al., 2017). In addition, *in situ* immobilization of lysine decarboxylase by phasin fusion on intracellular PHA was investigated and a stable and reusable form of lysine decarboxylase was produced (Seo et al., 2016). Although whole-cell biosynthesis of cadaverine is efficient, the high cost of the precursor L-lysine limits its further industrial application.

As a model strain for lysine production, *E. coli* has the advantages of fast growth rate and clear genetic background, and thus it could be used for cadaverine production (Park et al., 2007; Qian et al., 2009). Using recombinant *E. coli* under optimized culture conditions, 134.9 g/L L-lysine has been obtained with a productivity of 1.9 g/L/h (Ying et al., 2014). However, the efficiency of cadaverine production from glucose by *E. coli* through direct fermentation was extremely low: only 9.6 g/L with a productivity of 0.32 g/L/h (Qian et al., 2011). *E. coli* could not efficiently convert glucose to cadaverine even after overexpression of lysine decarboxylase, which might be due to the inhibition of endogenous cadaverine on the fermentation strain by binding to the membrane porin to cause inadequate cell absorption of nutrients (Delavega and Delcour, 1995; Iyer and Delcour, 1997).

Microbial consortia production might be an efficient strategy to mitigate toxicity and improve efficiency compared to the direct fermentation process for producing cadaverine from glucose using a single strain. Overexpression of L-lysine decarboxylase is beneficial when glycerol is used as the carbon source (Ma et al., 2015b); in contrast, glucose is the preferred source for the L-lysine producer *E. coli* NT1004 because of the glucose effect (Martinez et al., 2008; Martinez-Gomez et al., 2012). To avoid competition between the two strains utilizing the same carbon source, the glucose consumption pathway of the cadaverine bioconversion strain was removed when *E. coli* MG1655 was used as the starting strain.

**FIGURE 1:**
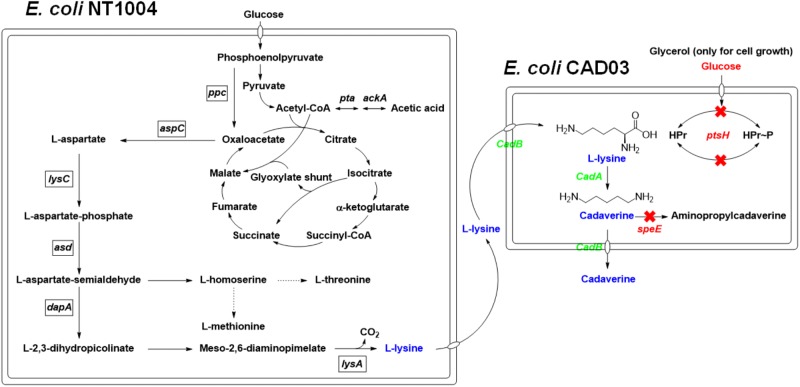
The overview of the pathways involved in this cadaverine bio-production. Overview of the pathways involved in the cadaverine production from glucose and glycerol in two engineered *E. coli*. The arrows represent up-regulated pathway. The dotted lines represent repression. The red X’s indicate that the genes are knocked out. Gene knockouts are indicated in red italics, and overexpressed genes are indicated in green italics. Enzymes encoded by the genes shown are: *ackA*, acetate kinase; *asd*, aspartate semialdehyde dehydrogenase; *aspC*, PLP-dependent _L_-aspartate aminotransferase, *cadA*, L-lysine decarboxylase; *cadB*, cadaverine-lysine antiporter; *dapA*, dihydrodipicolinate synthase; *lysA*, diaminopimelate decarboxylase; *lysC*, aspartate kinase III; *ppc*, phosphoenolpyruvate carboxylase; *pta*, phosphate acetyltransferase; *ptsH*, phosphohistidine carrier protein; *speE*, putrescine/cadaverine aminopropyltransferase.

In the present study, a microbial consortium for cadaverine production based on coupling whole-cell bioconversion of cadaverine and microbial fermentation of L-lysine by recombinant *E. coli* was evaluated. As shown in **Figure 1**, two strains, including an L-lysine producer using a glucose as carbon source and a cadaverine bioconversion strain with glucose metabolism deficiency using a glycerol as carbon source, were constructed. The glucose metabolism deficiency was achieved by modifying the PTS^Glc^ system of the cadaverine producer with CRISPR-Cas. By co-culturing the two strains, a novel microbial consortium process using a mixture of glucose and glycerol as carbon sources for cadaverine production was developed. This process had a higher production and efficiency than production with a single strain.

## Materials and Methods

### Construction of Strains and Plasmids

The bacterial strains and plasmids used in this study are summarized in **Supplementary Table S1**. *E. coli* NT1003 (Ying et al., 2014) was stored in the laboratory and used for high production of L-lysine. The PCR primers for plasmid constructions are listed in **Supplementary Table S2**. DNA manipulations were performed according to standard protocols. The plasmids pTrc99A (Mendez-Perez et al., 2017) and pET28a-*pelB*-CadBA were used as expression vectors, and DNA sequencing was performed by Genewiz (Suzhou, China).

The fragments Trc-4A and *PelB*-CadBA-4A were amplified by PCR using the primer Trc99A-4A-F/Trc99A-4A-R and *PelB*-CadBA-4A-F/*PelB*-CadBA-4A-R, with plasmid pTrc99A and pET28a-*pelB*-CadBA as the template, respectively. Subsequently, plasmid pTrc99A-*pelB*-CadBA was constructed by splicing the fragments Trc-4A and *PelB*-CadBA-4A using ClonExpress, and transformed into NT1003 competent cells to construct the strain CAD01 for direct cadaverine fermentation.

The *ptsG, ptsH, ptsI, glk, crr*, and *speE* genes of *E. coli* MG1655 were knocked out by CRISPR-Cas system (Jiang et al., 2013, 2015). The primers used for gene knockout (**Table 1**) were designed with the help of Guide RNA Target Design Tool^1^, and the specific steps are described in **Supplementary Methods**.

**Table 1:** Primers used for gene knockout.

Target gene	Gene knockout primers	Fragment 1 primers	Fragment 2 primers	Fragment fusion primers	Sequencing primers
*glk*	Target-Glk-F	glk-cas-F1	glk-cas-F2	glk-cas-F1	glk-CK-F
	T-crRNA-R	glk-cas-R1	glk-cas-R2	glk-cas-R2	glk-CK-R
*pts*G	Target-ptsG-F	PtsG-KO-F1	PtsG-KO-F2	PtsG-KO-F1	ptsG-KO-CK-F
	T-crRNA-R	PtsG-KO-R1	PtsG-KO-R2	PtsG-KO-R2	ptsG-KO-CK-R
*pts*H	Target-ptsH-F	ptsH-Cas-F1	ptsH-cas-F2	ptsH-Cas-F1	ptsH-KO-CK-F
	T-crRNA-R	ptsH-cas-R1	ptsH-cas-R2	ptsH-cas-R2	ptsH-KO-CK-R
*pts*I	Target-ptsI-F	ptsI-cas-F1	ptsI-cas-F2	ptsI-cas-F1	ptsI-KO-CK-F
	T-crRNA-R	ptsI-cas-R1	ptsI-cas-R2	ptsI-cas-R2	ptsI-KO-CK-R
*crr*	Target-crr-F	crr-cas-F1	crr-cas-F2	crr-cas-F1	crr-KO-CK-F
	T-crRNA-R	crr-cas-R1	crr-cas-R2	crr-cas-R2	crr-KO-CK-R
*spe*E	Target-speE-F	speE-cas-F1	speE-cas-F2	speE-cas-F1	speE-CK-F
	T-crRNA-R	speE-cas-R1	speE-cas-R2	speE-cas-R2	speE-CK-R

The strains MG1655 with knocked out *ptsG, ptsH, ptsI, glk*, and *crr* genes were named as MG1655G, MG1655H, MG1655I, MG1655K, and MG1655R, respectively. The *ptsH* and *speE*-knockout strain was named as MG1655HE (**Supplementary Table S1**). The plasmid pTrc99A-*pelB*-CadBA was transformed into strains MG1655H and MG1655HE to construct cadaverine producers CAD02 and CAD03, respectively. The plasmid pTrc99A was transformed into strain NT1003 to construct strain NT1004.

### Glucose and Glycerol Utilization Assays With Recombinant Strains

Knockout strains constructed were cultured at 37°C and 250 rpm in M9 medium supplemented with 5 g/L glucose or 5 g/L glycerol. After 12 h, the OD_600_ of each strain was examined. The strains CAD02, NT1004, and CAD02/NT1004 were cultured at 37°C and 250 rpm in M9 medium supplemented with 2.5 g/L glucose and 2.5 g/L the glycerol. The OD_600_, concentrations of glucose and glycerol and fermentation products for each experiment group were examined during the cultivation period. Each gene knocking-out experiment was carried out in triplicate using three gene-knockout strains.

### Cadaverine Fermentation Using a Single Strain or Microbial Consortia

The medium used for cadaverine fermentation with a single strain contained the following (g/L): Na_2_HPO_4_⋅12H_2_O, 17.1; KH_2_PO_4_, 3; NH_4_Cl, 10; KCl, 0.5; sodium pyruvate, 0.5; peptone, 0.6; betaine, 2; MgSO_4_, 1.6; FeSO_4_⋅7H_2_O, 0.032; MnSO_4_⋅H_2_O, 0.032; ZnSO_4_⋅7H_2_O, 0.086; CuSO_4_⋅5H_2_O, 0.077; L-Threonine, 0.3; L-Methionine, 0.1; glucose, 10; thiamine, 0.02; biotin, 0.002; nicotinamide, 0.01; and ampicillin (Amp), 0.1. The feeding medium for this process contained the following (g/L): glucose, 400; and NH_4_Cl, 400. The medium used for cadaverine fermentation with a microbial consortium was the same as mentioned above except for addition of 10 g/L glycerol. The feeding medium for this process was also the same as mentioned above except for the addition of 400 g/L glycerol.

Cadaverine fermentation using a single strain or microbial consortia was conducted in a 7.5-L fermenter (BioFio 115, New Brunswick Scientific, Edison, NJ, United States) with a liquid loading of 4 L. Seed cultures of strain CAD01, NT1004, CAD02, and CAD03 were incubated for 7–9 h in 100-ml Luria-Bertani (LB) broth at 37°C and 250 rpm. After achieving an OD_600_ of 5, 10% (v/v) seed cultures of CAD01, NT1004/CAD02 and NT1004/CAD03 were, respectively, inoculated into the fermenter under the conditions of 37°C, 300 rpm, and 1 vvm. Upon reaching an OD_600_ of 2, the cells were induced using 0.5 mM Isopropyl β-D-Thiogalactoside (IPTG), and then using 10 M ammonium hydroxide solution and 6 M hydrochloric acid to maintain the pH at 6.8. The dissolved oxygen (DO) content was monitored by using a Mettler oxygen electrode and maintained at about 10% by automatically adjusting the agitation speed. Residual glucose concentration was monitored offline and when the glucose level was less than 5 g/L, the feeding medium was added to maintain the glucose concentration about 5 g/L. All the experiments were carried out in triplicate.

### Optimization of Culture Conditions for Cadaverine Production Using Microbial Consortia

The optimization experiments were performed in shake flasks, and the fermentation medium used here was described as the medium used for cadaverine fermentation using microbial consortia. The strains NT1004 and CAD03 were inoculated into a shake flask at 1% (v/v), and incubated at 37°C and 250 rpm for 7–9 h. Subsequently, the cells were induced using 0.5 mM IPTG. For ascertaining the optimal initial inoculum ratio, the *E. coli* NT1004:CAD03 inoculum ratios of 1:1, 2:1, 4:1, 8:1, 10:1, and 15:1 were, respectively, employed. To optimize the initial sugar:glycerol ratio, an initial sugar : glycerol ratio gradient of 1:1, 2:1, 4:1, 6:1, 8:1, and 10:1 was used. For determining the optimal induction period, induction time of 3, 6, 9, 12, and 15 h were employed after co-culturing the mixed strains at a temperature gradient of 25, 30, 37, 42, and 47°C, respectively. For optimizing the DO content, a DO gradient of 15, 20, 25, 30, and 35% was used, respectively. For screening of optimal inorganic nitrogen source, three inorganic nitrogen sources, namely, (NH_4_)_2_SO_4_, NH_4_Cl, and urea, with the same mole quality of 0.076 M were investigated. The initial concentration of the optimum nitrogen source was optimized by employing concentrations of 2, 5, 10, 20, and 30 g/L, respectively. For ascertaining the optimal C/N ratio, the C/N ratios of 9:1, 6:1, 3:1, 3:2, 3:4, and 3:8 were used, respectively. Each optimization experiment was carried out in triplicate using strains NT1004/CAD03.

### Feeding Strategy for Cadaverine Production Using Microbial Consortia

The experiments were conducted in a 7.5-L fermenter with the same medium and conditions used in the microbial consortia cadaverine fermentation, except for the difference of feed-medium. The feed-medium used here is a mixture of 400 g/L glucose, 400 g/L glycerol and 400 g/L (NH_4_)_2_SO_4_ with a sugar:glycerol ratio of 8:1 and a C/N ratio of 3:2. According to the constant-rate feeding strategy, feeding rates of 5, 9, and 13 ml/h, were employed, respectively. At the end of cultivation, dry cell weight (DCW), cadaverine production, glucose consumption, and cadaverine productivity were determined. Each experiment was carried out in triplicate using strains NT1004/CAD03.

### Analytical Methods

The DCW was computed from a curve of OD_600_ with respect to dry weight. An OD_600_ of 1.0 represented 400 mg dry weight/L. The concentrations of glucose and L-lysine were analyzed by using an SBA-40C biosensor analyzer (Saurina et al., 1999) (Shandong Province Academy of Sciences, China). Acetic acid was evaluated by using high performance liquid chromatography (HPLC) (1290, Agilent Technologies, Santa Clara, CA, United States) equipped with an ion-exchange column (prevail organic acid 5 μ, 250 × 4.6 mm, Grace, Columbia, MD, United States) and 25 mM KH_2_PO_4_ (adjusted to a pH of 2.5 by H_3_PO_4_) was used as a mobile phase with a flow rate of 1 mL/min (Ying et al., 2014). Cadaverine was detected by HPLC after derivatization with dansyl chloride, using an Agilent 1290 Infinity System (Santa Clara, CA, United States) equipped with a fluorescence detector (FLDG1321B) (Ma et al., 2015b). The specific steps are described in **Supplementary Methods**.

## Results

### Constructing the Glucose Metabolism Deficient Strain With CRISPR-Cas

**FIGURE 2:**
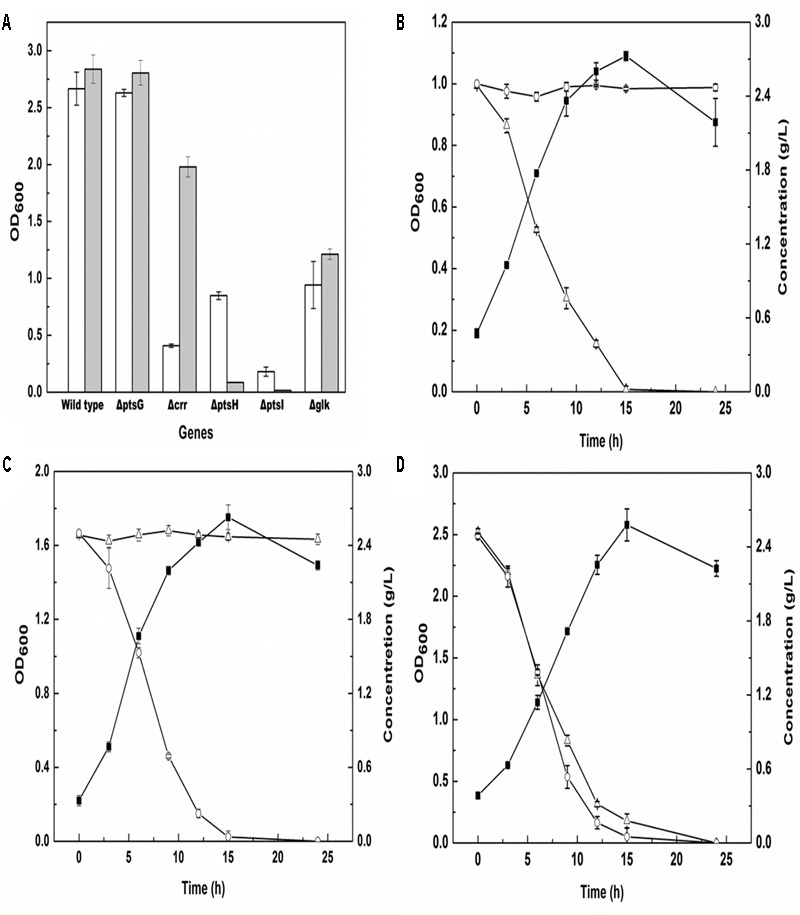
Gene screening for glucose metabolism deficiency and carbon sources utilization of engineered *E. coli*. **(A)** OD_600_ of *E. coli* MG1655 and four PTS^Glc^ mutants in the M9 medium supplied with 5 g/L glycerol (unfilled bar) or 5 g/L glucose (filled bar) after 12 h cultivation; **(B–D)** OD_600_ (filled squares), glucose (open triangles) and glycerol (open circles) consumptions of NT1004 **(B)**, CAD03 **(C)** and mixture strains (NT1004 and CAD03) **(D)** in the M9 medium supplied with 2.5 g/L glycerol and 2.5 g/L glucose during 24 h cultivation. Results are means of triplicate experiments, and error bars represent ± standard deviation (SD).

The enzymes involved in the PTS^Glc^ system and encoded by genes *ptsG, ptsH, ptsI, crr*, and *glk* (Xia et al., 2012) were knocked out to achieve glucose metabolism deficiency. The genes *ptsG, ptsH, ptsI, crr*, and *glk* encode enzyme IICB^Glc^, the phosphohistidine carrier protein, enzyme I, IIA^Glc^ protein and glucokinase, respectively. The knockout manipulation was performed on the *E. coli* MG1655 strain using the CRISPR-Cas system, and the growth of the knockout strains in glucose and glycerol was examined. As shown in **Figure 2A**, there were no significant differences in glucose and glycerol utilization when the *ptsG* or *glk* gene was knocked out, whereas knockout of the *crr* gene led to a significant decrease in cell growth when glycerol was used as the carbon source, and the biomass was 4.8-fold lower than that of the strain cultured in glucose. Knockout of the *ptsI* gene meant that the strain could not grow in both carbon sources. Furthermore, knockout of the *ptsH* gene resulted in the inability to grow in the presence of glucose and in a 1/3 decrease in biomass in the presence of glycerol, when compared with the wild-type strain. Furthermore, spermidine synthase, encoded by the gene *speE* and responsible for cadaverine degradation (Cacciapuoti et al., 2007), was knocked out in the strain MG1655H using CRISPR-Cas to obtain a new strain MG1655HE.

### Identifying the Consortium Utilization of Carbon Sources for Cadaverine Production

To optimize the microbial consortium fermentation process to eliminate bottlenecks in cadaverine fermentation with a single strain, the consortium utilization of carbon sources was investigated. First, the recombinant strains were constructed. *E. coli* NT1003 was used as the L-lysine-producing host strain. To facilitate growth in the presence of Amp, plasmid pTrc99A was transformed into *E. coli* NT1003 to obtain *E. coli* NT1004. For direct cadaverine fermentation from glucose, cadaverine-producing *E. coli* CAD01 was constructed by transforming the plasmid pTrc99A-*pelB*-CadBA, containing L-lysine decarboxylase and lysine/cadaverine antiporter (CadB) with N-terminal fused *pelB* signal peptide, into NT1003. For microbial consortium fermentation, the plasmid pTrc99A-*pelB*-CadBA was transformed into the *ptsH*-knockout strain MG1655H to generate cadaverine-producing CAD02. Furthermore, the plasmid pTrc99A-*pelB*-CadBA was transformed into MG1655HE to generate cadaverine-producing CAD03.

To ascertain the utilization selectivity of the engineered strains on different sugars, each strain was cultivated in M9 medium containing glucose and glycerol as mixed carbon sources. As shown in **Figures 2B–D**, all strains successfully exhausted their target carbon sources without consuming another sugar. Furthermore, the growth of two strains in the same medium was examined and the results showed that the consumption rate of glucose and glycerol was similar to that of single strain cultivation. The increase in cell density of the consortium represented the total biomass of both strains. In addition, the fermentation products of the two strains were also detected, and no cadaverine or L-lysine were observed in NT1004 or CAD01 fermentation broth, respectively (data not shown).

### Identifying the Microbial Consortium Fermentation Process for Cadaverine Production

**FIGURE 3:**
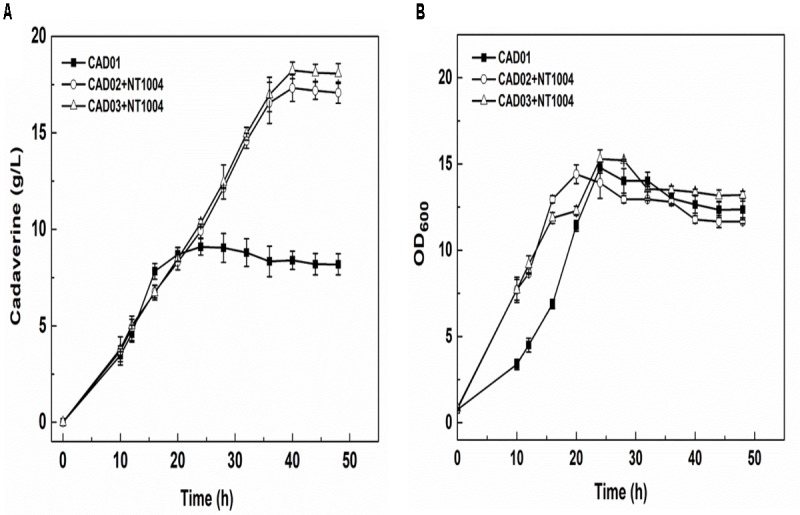
Assays of cadaverine fermentation using different strains. **(A)** Cadaverine production of CAD01, CAD02/NT1004, and CAD03/NT1004 microbial consortia; **(B)** Biomass of CAD01, CAD02/NT1004, and CAD03/NT1004 microbial consortia. Three different strains were induced using 0.5 mM IPTG when reaching an OD_600_ of 2. Results are means of triplicate experiments, and error bars represent ± standard deviation (SD).

After strain construction and the sugar utilization test, cadaverine fermentation using CAD01, CAD02/NT1004, and CAD03/NT1004 microbial consortia was performed, and the amount of cadaverine produced as well as productivity were determined. As shown in **Figure 3A**, 17.1 ± 0.5 g/L cadaverine was obtained after 48 h of fermentation using microbial consortium, which was 2.1-fold higher than that obtained using CAD01 alone. In addition, the highest cadaverine productivity reached 0.61 g/L/h, which is 1.9-fold higher than that previously reported (0.32 g/L/h) (Qian et al., 2011). As shown in **Figure 3B**, compared with the microbial consortia, a growth delay was observed in strain CAD01, which might be caused by the plasmid burden since CAD01 harbored two plasmids while the two microbial consortia harbored a plasmid in each strain. Moreover, there was no obvious difference in the results obtained with CAD03/NT1004 and CAD02/NT1004 (*p* = 0.898, α = 0.05); further experiments were performed using the strain CAD03 to ensure accuracy.

### Optimization of the Culture Conditions for Improved Cadaverine Production Using a Microbial Consortium

#### Effect of Inoculation Method and Induction Conditions

**FIGURE 4:**
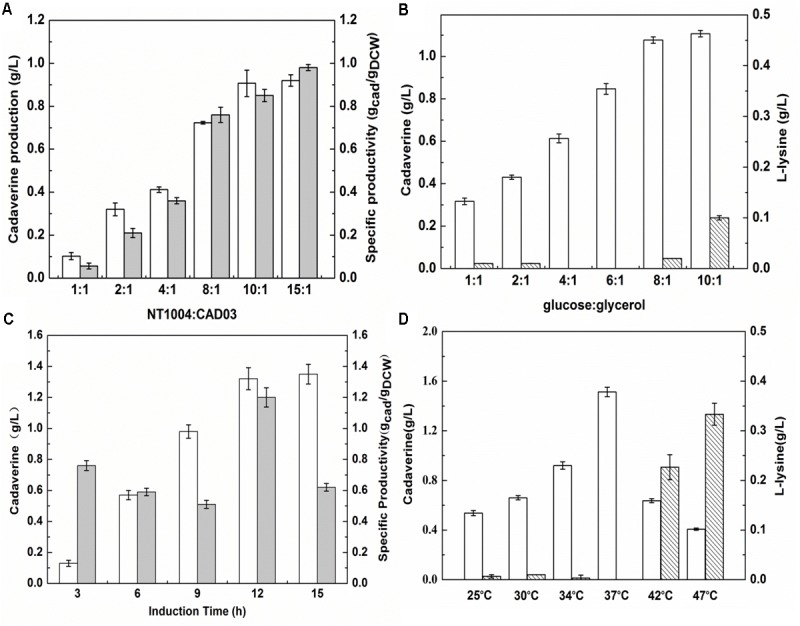
Optimizing the inoculation and induction conditions for cadaverine fermentation using microbial consortia. **(A)** The optimum inoculation ratio of NT1004/CAD03; **(B)** The optimum initial ratio of glucose/glycerol; **(C)** The optimum induction time; **(D)** The optimum induction temperature. The unfilled bar represents cadaverine production (g/L), the filled bar represents cadaverine specific productivity (gcad/gDCW), the bar with backslash represents L-lysine production (g/L). Results are means of triplicate experiments, and error bars represent ± standard deviation (SD).

During the microbial consortium fermentation process, the different growth rates and productivity of two strains could lead to an insufficient or excessive supply of L-lysine and an unsatisfactory amount of cadaverine production. To balance the growth rate of a synthetic two-member microbial consortium, in the present study, the initial inoculum ratio and sugar:glycerol ratio were optimized. Furthermore, as the cadaverine catalyst, the activity of overexpressed L-lysine decarboxylase is important to efficiently convert L-lysine (Ma et al., 2015b). Hence, the induction time and temperature were further optimized to investigate their effect on cadaverine production. As shown in **Figure 4A**, based on a range of 1:1 to 15:1, cadaverine production peaked at the NT1004:CAD03 inoculum ratio of 10:1. Cadaverine production increased with increasing glucose concentration in the medium (**Figure 4B**). However, when the glucose:glycerol ratio in the medium was 8:1, the produced L-lysine remained in the medium and its concentration increased with further increase in glucose content. These results indicated that the produced L-lysine was in excess of the demand of lysine decarboxylase when the inoculum ratio and glucose:glycerol ratio were higher than 10:1 and 8:1, respectively, which could result in wasting L-lysine. As shown in **Figure 4C**, cadaverine production increased with the induction time from 3 to 12 h; however, no significant increase in cadaverine production was observed after 12 h and the optimal induction time was observed when IPTG was added at 9 h of cultivation. **Figure 4D** illustrates that the optimum cadaverine production was obtained at 37°C, which might be due to the unsatisfactory enzyme expression at low temperature and the decreased enzyme activity at high temperature. Under optimal conditions, with leftover L-lysine concentration of 0.02 g/L, the cadaverine production reached 1.53 g/L, which was 13.3% higher than that obtained before optimization.

#### Effect of DO Content

**FIGURE 5:**
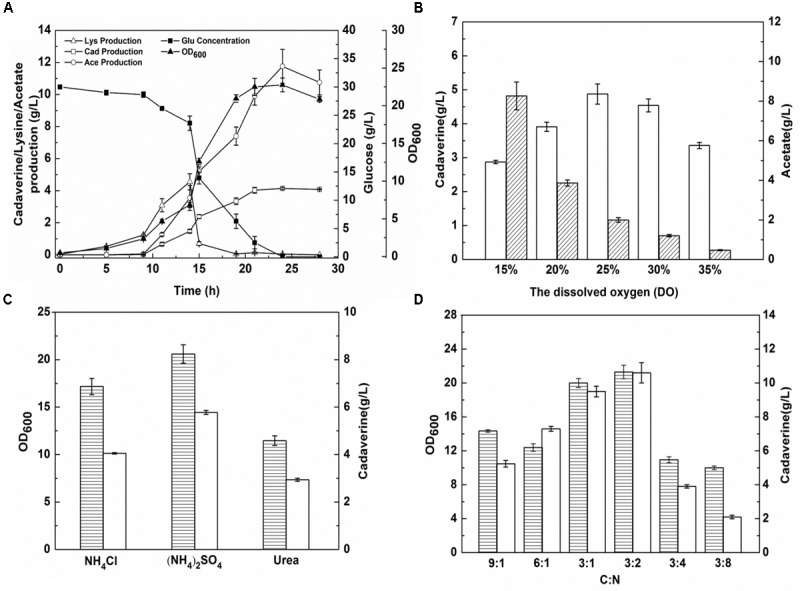
The effects of DO content and nitrogen source on cadaverine production using microbial consortia. **(A)** The production of cadaverine using recombinant strains NT1004 and CAD03; **(B)** The optimum DO content; **(C)** The optimum nitrogen source; **(D)** The optimum C/N ration. The unfilled bar represents cadaverine production (g/L), the bar with forward slash represents acetate production (g/L), the bar with horizontal lines represents OD_600_. Results are means of triplicate experiments, and error bars represent ± standard deviation (SD).

*Escherichia coli* produces acetic acid when the level of DO in the culture medium is insufficient (Suarez and Kilikian, 2000). Before DO content optimization, cadaverine fermentation using the microbial consortium was performed in a 1.4-L cascade fermenter with 30 g/L glucose and 4 g/L glycerol as the carbon sources. As shown in **Figure 5A**, four stages were observed during the fermentation process and there was a low level of cadaverine production with high acetic production. To optimize DO content, a DO gradient from 15 to 35% was employed to improve cadaverine production. As shown in **Figure 5B**, acetic acid production decreased with the increase in DO content. Furthermore, cadaverine production increased with the increasing DO level from 15 to 25%, but decreased at excessive amounts of DO (>25%). Therefore, the optimal DO content was 25% at which cadaverine and acetic acid production reached 4.78 and 2 g/L, respectively, which were 1.17- and 0.2-fold higher than those achieved before optimization, respectively.

#### Effect of Nitrogen Source

To further increase L-lysine and cadaverine production, other conditions in the fermenter were optimized. The effects of (NH_4_)_2_SO_4_, NH_4_Cl, and urea (0.076 M each) on cell growth and product synthesis were investigated. As shown in **Figure 5C**, better cell growth and higher cadaverine production were observed when (NH_4_)_2_SO_4_ was used as the nitrogen source, with OD_600_ and cadaverine titer reaching 20.6 and 5.77 g/L, respectively. Subsequently, the effect of different concentrations of (NH_4_)_2_SO_4_ on cadaverine production was investigated. Cadaverine production peaked at the (NH_4_)_2_SO_4_ concentration of 5 g/L with a titer of 7.86 g/L (**Table 2**). During the production process, the C/N ratio also exerted significant effects on cell growth and production (Shiloach et al., 1996). To optimize of the C/N ratio, a C/N ratio gradient from 9:1 to 3:8 was employed. As shown in **Figure 5D**, both OD_600_ and cadaverine production increased with decreasing C/N ratio, and then decreased significantly. This might be due to excessive ammonium ions inhibiting cell growth and L-lysine production (Ying et al., 2014). The optimal C/N ratio was 3:2, which resulted in a cadaverine titer of 10.6 g/L, which was 1.35-fold higher than that achieved before optimization.

**Table 2:** The effects of different concentrations of (NH_4_)_2_SO_4_ on cadaverine production using microbial consortia.

Fermentation parameters	Initial concentration of (NH_4_)_2_SO_4_ (g/L)				
	
	2	5	10	20	30
Glucose (g/L)	30.2 ± 0.58	31 ± 0.25	25 ± 0.4	18 ± 0.21	12 ± 3.19
Time (h)	24	24	24	24	24
DCW (g/L)	8.7 ± 0.16	9.68 ± 0.71	6.35 ± 1.51	3.54 ± 0.29	1.69 ± 0.32
Cad (g/L)	6.2 ± 0.32	7.8 ± 0.64	5.53 ± 0.17	2.14 ± 0.09	1.05 ± 0.007
Productivity (g/L/h)	0.26 ± 0.012	0.33 ± 0.013	0.23 ± 0.007	0.09 ± 0.001	0.04 ± 0.001

*Results are means of triplicate experiments, and are represented as ± standard deviation (SD).*

### Feeding Strategy for Improved Cadaverine Production Using Microbial Consortia

L-lysine, a basic amino acid, has two amino groups, indicating that two moles of ammonium are required to produce 1 mol of L-lysine (Ying et al., 2014). As ammonium in the culture medium is not sufficient for the production of L-lysine, addition of feed medium containing mixed carbon sources and (NH_4_)_2_SO_4_ to the medium is necessary. As mentioned earlier, a high concentration of ammonium sulfate inhibits both cell growth and L-lysine production. Therefore, in the present study, constant and variable speed (5, 9, 13 ml/h, respectively) feeding strategies were employed. During these three microbial consortium fermentation processes, although the DCW, cadaverine production, glucose consumption, and cadaverine productivity increased with increasing flow rate, a carbon-nitrogen source excess was observed at a flow rate of 13 ml/h (data not shown).

**FIGURE 6:**
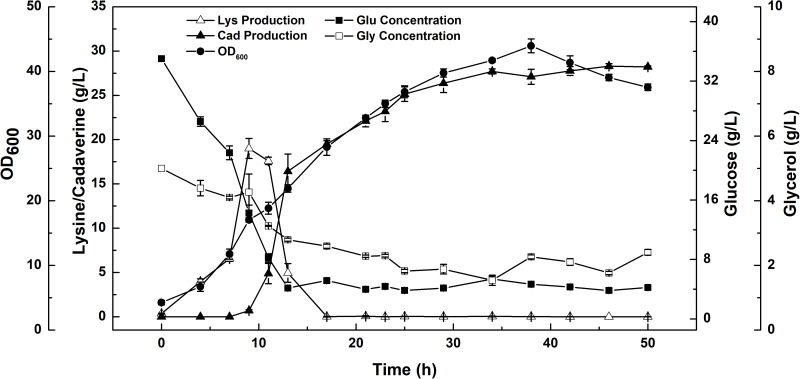
The enhanced production of cadaverine from glucose using a consortium of two engineered *E. coli* NT1004 and CAD03. Results are means of triplicate experiments, and error bars represent ± standard deviation (SD).

A constant-rate feeding strategy could not significantly enhance cadaverine production (**Table 3**). Therefore, a multi-stage constant-speed feeding strategy was employed to improve cadaverine production. This feeding strategy was divided into three stages: feeding at 15, 25, and 35 h and at flow rates of 9, 13, 5 ml/h, respectively. To investigate the cell growth and nutrient utilization, the strain ratio over culture time was monitored (**Supplementary Figure S1**). In the early stage, the initial carbon and nitrogen sources were consumed, the two strains grew almost simultaneously and the growth rate was slow, and hence, the feeding flow rate was 9 ml/min. Subsequently, when the strain growth was in the logarithmic phase, the feeding flow rate was increased to 13 ml/min to meet the carbon and nitrogen sources requirements for growth. However, in this stage, the growth rate of CAD03 decreased due to the expression of decarboxylase. At the end of the fermentation period, the growth of the strain began to stagnate and the strain ratio was stabilized, and hence, the feed flow rate was reduced to 5 ml/min to avoid wasting carbon and nitrogen. Using a multi-stage constant-speed feeding strategy, after fermentation of 50 h, the final cadaverine amount reached 28.5 g/L, with a molar yield of 0.209 g/g (**Figure 6**), which was 1.6- and 3-fold higher, respectively, than that obtained before optimization and compared to values reported in the literature (Qian et al., 2011).

**Table 3:** The effects of different constant flow rates on the microbial consortia cadaverine fermentation.

Fermentation parameters	Different feeding flow rates		
	
	V_1_ = 5 ml/h	V_2_ = 9 ml/h	V_3_ = 13 ml/h
Glucose (g/L)	58 ± 1.18	82.3 ± 9.43	110.2 ± 8.25
Time (h)	48	48	48
DCW (g/L)	12.3 ± 0.15	16.2 ± 2.19	20 ± 3.35
Cad (g/L)	11.25 ± 0.16	16.3 ± 0.81	20.97 ± 0.38
Productivity (g/L/h)	0.26 ± 0.002	0.34 ± 0.011	0.44 ± 0.005

*Results are means of triplicate experiments, and are represented as ± standard deviation (SD).*

## Discussion

Production of chemicals from renewable resources has received increased attention recently due to environmental concerns and limited resources of fossil fuels. Cadaverine is an important platform chemical, particularly as a building block for synthesizing PA 5X with dicarboxylic acid. Since microbial fermentation of succinic acid and castor oil extraction of sebacic acid have been thoroughly developed (Lee et al., 2002; McKinlay et al., 2005), bio-based production of cadaverine is highly desirable for production of bio-based nylons. In this paper, we reported the development of microbial consortium fermentation of two recombinant *E. coli* strains that efficiently produces cadaverine.

Previous studies showed that the whole-cell bioconversion is a common method for the production of cadaverine (Ma et al., 2015b). However, the high cost of the precursor L-lysine limits further industrial application of this method. Theoretically, *E. coli* is a potential cadaverine producer because it contains L-lysine decarboxylase, CadA, responsible for a biosynthesis pathway converting an L-lysine to cadaverine, and the degradation and utilization pathways of cadaverine are relatively well studied. The *E. coli* Cad system is used to protect cells against acid stress by inducing protein expression under conditions of low pH with excess L-lysine (Moreau, 2007). Two principal components were involved in the *E. coli* Cad system: the *cadBA* operon coding for lysine decarboxylase (CadA) and lysine/cadaverine antiporter (CadB), and the regulatory protein CadC. With accumulation of cadaverine up to 235 μM, excess cadaverine would function as a negative effector of *cadBA* expression by deactivation of CadC through binding to the site at the dimerization interface of CadC (Neely and Olson, 1996; Haneburger et al., 2012). In addition, CadA activity is inhibited by *ppGpp*, a stringent response effector that accumulates rapidly in cells that are starved for amino acids, to prevent excessive L-lysine consumption (Kanjee et al., 2011). Therefore, a low efficiency of cadaverine production by *E. coli* through direct fermentation was observed even after overexpression of L-lysine decarboxylase.

Microbial consortium production is an efficiency strategy to complete tasks that would be too difficult for one organism, and it has broad applications for producing nutraceuticals, drugs, and biofuels, and in medicine and human health, and environments (Song et al., 2014). To efficiently produce cadaverine, two metabolically engineered *E. coli* strains responsible for L-lysine synthesis and cadaverine conversion were used simultaneously. For cadaverine production using this microbial consortium, the carbon source metabolic pathway of the strains was initially modified to avoid competition between the two strains on the same carbon source. Therefore, in the present study, five functional proteins involved in the phosphotransferase system, the most efficient system responsible for glucose transport in *E. coli* (Liang et al., 2015), were knocked out using the CRISPR-Cas9 method. Our results and observation were consistent with previous studies (Liang et al., 2015). The entire *ptsHI-crr* operon servers two common steps involved in the PEP: carbohydrate phosphotransfer cascades. EI and HPr are the common transporter proteins in all PTSs. Therefore, inactivating EI (strain MG1655I) and HPr (strain MG1655H) should entail recruiting a non-PTS transport system such as the glycerol system. The experimental results support this assumption (**Figure 2A**). A striking effect in MG1655I indicated that the protein EI, which plays a vital role in physiological signaling (Cases et al., 2007; Doucette et al., 2011), autophosphorylation and the subsequent phosphotransfer reaction are the limiting steps in the phosphotransfer cascade of the PTS^Glc^ (Gabor et al., 2011). Although the IIA^Glc^ and IICB^Glc^ are glucose-specific components, their inactivation mutants MG1655C and MG1655G could recruit both PTSs and non-PTSs. This consortia engineering was successfully applied to other works. Xia et al. obtained succinate from xylose-glucose mixtures successfully using a consortium of two engineered *E. coli* strains, with glucose and xylose metabolism deficiencies, respectively (Xia et al., 2015).

The direct fermentation of cadaverine using single strain CAD01 was also performed in the present study and the results showed that the final cadaverine production was only 9.09 ± 0.43 g/L after 20 h of cultivation, followed by a stagnation, which is consistent with the fermentative production of cadaverine by strain XQ56 (Qian et al., 2011). When using microbial consortia, a final cadaverine production of 28.5 g/L with a molar yield of 0.209 g/g was obtained. Compared with the studies by Qian et al. (2011), our cadaverine production and productivity were 3-fold and 2.7-fold higher than those of strain XQ56. Our results indicated that two separate processes might improve cell growth and process efficiency. During the microbial consortium process, the cadaverine was produced by efficient bioconversion using a biocatalyst strain with overexpression of L-lysine decarboxylase, which might enhance the productivity of cadaverine. Furthermore, the supply of L-lysine was also improved due to the release of the inhibition of high intracellular cadaverine concentration on the growth and activity of the L-lysine producer. It has been reported that cadaverine can bind to the membrane porins OmpC and OmpF, resulting in membrane porin closure and causing inadequate cell absorption of the culture medium and exclusion of harmful intracellular metabolites (Delavega and Delcour, 1995). It has also been demonstrated that the ability of extracellular cadaverine to close membrane porin is weaker than that of intracellular cadaverine (Iyer and Delcour, 1997). In the present study, *E. coli* NT1004 was only used to produce L-lysine and *E. coli* CAD03 was only used as a biocatalyst to convert L-lysine to cadaverine. For whole-cell bioconversion, a small amount of cell biomass was needed for cadaverine bioconversion due to the high activity of decarboxylase. The production of cadaverine would not be affected because only a small amount of nutrients was needed for cell activity of the cadaverine catalyst and the L-lysine producer NT1004 could not be inhibited by extracellular cadaverine. Thus, the use of this microbial consortium for cadaverine production could significantly reduce membrane porin closure and prevent growth inhibition caused by high intracellular cadaverine concentration, as well as enhance cadaverine production, which suggested that two separate processes would eliminate the cadaverine fermentation bottleneck during production with a single strain.

When compared with direct fermentation using a single recombinant strain, the microbial consortium used in this study prevented the inhibition of cadaverine on cell growth and achieved a higher cadaverine titer. However, when compared with whole-cell conversion, although the use of an expensive substrate such as L-lysine was avoided in microbial consortium production, the cadaverine production and productivity were still not satisfactory, which could be related to the low supply rate of L-lysine and cell viability. Nevertheless, the fermentative cadaverine (28.5 g/L) obtained through *E. coli* fermentation in the present study is the highest reported so far and is 3-fold higher than that reported previously (Qian et al., 2011). The results of the present study might be useful to enhance the cadaverine production system from cheap carbon sources and the production and productivity we achieved could be further improved by enhancing L-lysine supply and cell viability.

## Author Contributions

PO, KC, XW, and SX designed the study. WM and HY provided instructions of the molecular biology experiments. JW performed majority work of this study. XL developed and performed the chemical analysis. JW wrote the manuscript. All authors read and approved the final manuscript.

## Conflict of Interest Statement

The authors declare that the research was conducted in the absence of any commercial or financial relationships that could be construed as a potential conflict of interest.

## Footnotes

1 http://chopchop.cbu.uib.no/

## Supplementary Material

FIGURE S1 The strain ratio during the culture time. The blue line represents OD_600_ of microbial consortia. Results are means of triplicate experiments, and error bars represent ± standard deviation (SD).

TABLE S1 Strains and plasmids used in this work.

TABLE S2 Primers used in this work.
