# Supplementary material for: A Novel Process for Cadaverine Bio-Production Using a Consortium of Two Engineered Escherichia coli
Source: Front Microbiol. 2018 Jun 19;9:1312. doi: 10.3389/fmicb.2018.01312 (PMC6018084; doi:10.3389/fmicb.2018.01312)
Supplement: TABLE S1 — Strains and plasmids used in this work. [file Table_1.DOCX]

Supplementary Tables

**Supplementary Table 1.** Strains and plasmids used in this work.

| Strains or plasmids | Description | Sources or Refs. |
| --- | --- | --- |
| Strains |  |  |
| *E.coli* NT1003 | *E.coli* W3110 lacI *thrB-* *metA-* | ([Ying et al., 2014](#_ENREF_3)) |
| *E.coli* NT1004 | NT1003 harboring pTrc99A: pTrc lacI + Amp^r^ | This work |
| CAD01 | NT1003 harboring pTrc99A-*pelB*-CadBA | This work |
| MG1655-cas | MG1655harboring pCas | This work |
| MG1655G | MG1655 Δ*ptsG* | This work |
| MG1655H | MG1655 Δ*ptsH* | This work |
| MG1655I | MG1655 Δ*ptsI* | This work |
| MG1655K | MG1655 Δ*glk* | This work |
| MG1655R | MG1655 Δ*crr* | This work |
| MG1655HE | MG1655 Δ*ptsH* Δ*speE* | This work |
| CAD02 | MG1655H harboring pAmp-Trc-pel-CadBA | This work |
| CAD03 | MG1655HE harboring pAmp-Trc-pel-CadBA | This work |
| Plasmids |  |  |
| pTrc99A | P_trc_ promoter, pBR322 origin, lacI^q^, Amp^r^ | ([Mendez-Perez et al., 2017](#_ENREF_2)) |
| pET28a-*pelB*-CadBA | *cadBA* and *pelB* fragment inserted between the *Bam*HI-*Not*I and *Bgl*II-*Nco*I sites of pET28a | ([Ma et al., 2015](#_ENREF_1)) |
| pTrc99a-*pelB*-CadBA | Trc-4A and PelB-CadBA-4A fragments assembly | This work |

# References

Ma, W., Cao, W., Zhang, H., Chen, K., Li, Y., and Ouyang, P. (2015). Enhanced cadaverine production from L-lysine using recombinant Escherichia coli co-overexpressing CadA and CadB. *Biotechnology Letters* 37(4)**,** 799-806. doi: 10.1007/s10529-014-1753-5.

Mendez-Perez, D., Alonso-Gutierrez, J., Hu, Q., Molinas, M., Baidoo, E.E.K., Wang, G., et al. (2017). Production of jet fuel precursor monoterpenoids from engineered Escherichia coli. *Biotechnology and Bioengineering* 114(8)**,** 1703-1712. doi: 10.1002/bit.26296.

Ying, H., He, X., Li, Y., Chen, K., and Ouyang, P. (2014). Optimization of Culture Conditions for Enhanced Lysine Production Using Engineered Escherichia coli. *Applied Biochemistry and Biotechnology* 172(8)**,** 3835-3843. doi: 10.1007/s12010-014-0820-7.
