# Supplementary material for: A Novel Process for Cadaverine Bio-Production Using a Consortium of Two Engineered Escherichia coli
Source: Front Microbiol. 2018 Jun 19;9:1312. doi: 10.3389/fmicb.2018.01312 (PMC6018084; doi:10.3389/fmicb.2018.01312)
Supplement: TABLE S2 — Primers used in this work. [file Table_2.DOCX]

Supplementary Tables

**Supplementary Table 2.** Primers used in this work.

| Primer | Sequence | Sources or Refs. |
| --- | --- | --- |
| Trc99a-4A-F | CATGCGAGAGTAGGGAACTGCC | This work |
| Trc99a-4A-R | GGTCTGTTTCCTGTGTGAAATTGTTATCC | This work |
| *PelB*-CadBA-4A-F | TTTCACACAGGAAACAGACCATGAAATACCTGCTGCCGACC | This work |
| *PelB*-CadBA-4A-R | CAGTTCCCTACTCTCGCATGTTAGTGGTGGTGGTGGTGGTGT | This work |
| T-crRNA-R | ACTAGTATTATACCTAGGACTGAGC | This work |
| Target-Glk-F | AATACTAGTGTATGCATTAGTCGGTGATGGTTTTAGAGCTAGAAATAGC | This work |
| glk-cas-F1 | ACGGAGTGGATGATGATGGTCG | This work |
| glk-cas-F2 | GTGACCTAAGGTCCATCACCGACTAATGCATACTTTGTCATTC | This work |
| glk-cas-R1 | TTAGTCGGTGATGGACCTTAGGTCACATTCTGTAAATCCTTCC | This work |
| glk-cas-R2 | GGGGTCTGGTCCGGCATGAC | This work |
| glk-CK-F | ACGGAGTGGATGATGATGG | This work |
| glk-CK-R | AGCGGTAGCAATGTGGTAT | This work |
| Target-ptsG-F | AATACTAGTCGACATTCCGCGTTATATGGGTTTTAGAGCTAGAAATAGC | This work |
| PtsG-KO-F1 | GGTTACTGGTGGAAACTGACTCACC | This work |
| PtsG-KO-F2 | GCACTCTCAATTATGTACATCCGTAACCACTAATCCGTAAGAC | This work |
| PtsG-KO-R1 | GTGGTTACGGATGTACATAATTGAGAGTGCTCCTGAGTATGG | This work |
| PtsG-KO-R2 | CAGTCAGTAAAGGGGTGGAATTTGAACT | This work |
| ptsG-KO-CK-F | ATGTTTTTAGTCGACTCACACTGCCA | This work |
| ptsG-KO-CK-R | GCCAACATCTTCCCGGATGAAATTG | This work |
| Target-ptsH-F | AATACTAGTCCTGTTTAAACTGCAGACTCGTTTTAGAGCTAGAAATAGC | This work |
| ptsH-Cas-F1 | GATGAAAGCTTTACCAACAAGAATATTGTGGTTATTC | This work |
| ptsH-cas-F2 | GGAAATACAATGGCGGAACTCGAGTAATTTCC | This work |
| ptsH-cas-R1 | ACTCGAGTTCCGCCATTGTATTTCCCCAACTTATAGGTTTAGTGTTGT | This work |
| ptsH-cas-R2 | GATAATCTTCAGGCCCAGGATGTTGC | This work |
| ptsH-KO-CK-F | CTGGCAGGTGAAGAGATTA | This work |
| ptsH-KO-CK-R | CACCTTCAACGTCACGAACCG | This work |
| Target-ptsI-F | AATACTAGTTCGTACTGAGTTCCTGTTCAGTTTTAGAGCTAGAAATAGC | This work |
| ptsI-cas-F1 | GGTTCAATTCTTCCTTTAGCGGCATAATG | This work |
| ptsI-cas-F2 | AGTAAGGTAGGGTTATGGAAAAAACAATCTGCTAATCCACGAGATGC | This work |
| ptsI-cas-R1 | TTAGCAGATTGTTTTTTCCATAACCCTACCTTACTTGTGACTGATTTT | This work |
| ptsI-cas-R2 | TGATTTCGTCCATGTTGGAGATAACAACC | This work |
| ptsI-KO-CK-F | CAGTAATGCCAGCTTGTTAAAAATGCG | This work |
| ptsI-KO-CK-R | CCAGCGCCTCCGGTAATGA | This work |
| Target-crr-F | AATACTAGTGTTGAACTGTTCGTCCACTTGTTTTAGAGCTAGAAATAGC | This work |
| crr-cas-F1 | TGAAATCGGCGTAATGGTGGAAACA | This work |
| crr-cas-F2 | AGGAGAAGATCATGATCCGCATCAAGAAGTAATTCTTGCC | This work |
| crr-cas-R1 | TTCTTGATGCGGATCATGATCTTCTCCTAAGCAGTAAATTGGG | This work |
| crr-cas-R2 | GTCAAACCTGACCTTCCCGAAGC | This work |
| crr-KO-CK-F | GCGATGGATCGTAGAGAGATCCTG | This work |
| crr-KO-CK-R | GGTTGGATTACGATGAGGA | This work |
| Target-speE-F | AATACTAGTTTTAAGCTGGTGATCGACGAGTTTTAGAGCTAGAAATAGC | This work |
| speE-cas-F1 | GCAGTGTGCTAACGTTTATCTTCTTTAAGC | This work |
| speE-cas-F2 | TCAACCCATGGCTTCACAGCCGTCCTAAG | This work |
| speE-cas-R1 | GGCTGTGAAGCCATGGGTTGATACCTCCTTTG | This work |
| speE-cas-R2 | AGCTGGTGGATCAGGTAATTCAGC | This work |
| speE-CK-F | CTCTTCCGTCAGTTGGTTGTAG | This work |
| speE-CK-R | CGCCTTCATATCGTCAGACATAA | This work |
