# Supplementary material for: A Novel Process for Cadaverine Bio-Production Using a Consortium of Two Engineered Escherichia coli
Source: Front Microbiol. 2018 Jun 19;9:1312. doi: 10.3389/fmicb.2018.01312 (PMC6018084; doi:10.3389/fmicb.2018.01312)
Supplement: Supplementary file 4 [file Presentation_1.PDF]

Supplementary Material

A novel process for cadaverine bio-production using a consortium of two engineered Escherichia coli

Jing Wang, Xiaolu Lu, Hanxiao Ying, Weichao Ma, Sheng Xu, Xin Wang, Kequan Chen*, Pingkai Ouyang

*** Correspondence: Kequan Chen**: kqchen@njtech.edu.cn

1 Supplementary Methods

1.1 Construction of gene knockout strain using CRISPR-Cas technology

A pTarget-X plasmid for the synthesis of the crRNA was constructed (X represents the corresponding target gene). PCR amplification was performed using the pTargetF plasmid (Supplementary Table 2) as a template using knockout primers (Table 1), and then the amplified product was digested with DpnI and transformed into *E. coli* Trans1-T1, culturing overnight. The three clones were picked and sent to GENEWIZ for plasmid sequencing by using the primers Target-CK-F and Target-CK-R (Appendix II). The strain containing the correct plasmid was cultured and the pTarget-X plasmid was extracted.

The genomic DNA of *E. coli* MG1655 was used as template to amplify the upstream and downstream fragments of the target gene using the fragment 1 primer and the fragment 2 primer in Table 1 respectively. Then, the upstream and downstream fragments of each gene were fused using fragment fusion primers by overlapping PCR methods to obtain a replacement fragment for knockout genes.

The plasmid pCas was transformed into *E. coli* MG1655 and cultured overnight on a solid LB medium containing 50 μg mL-1 of kanamycin at 30 ℃, and the cells were named MG1655-cas. The single clone MG1655-cas was inoculated in 5 mL LB liquid medium containing 50 μg mL^{-1} of kanamycin, incubated at 30 ℃ and 250 rpm for 9 h. Then 0.5 mL seed culture were inoculated into a 50 mL LB liquid medium containing 50μg mL^{-1} of kanamycin, and incubated at 30 ℃, 250rpm. Upon reaching an OD_{600} of 0.2, L-arabinose was added at a final concentration of 30 mM. When the OD_{600} was about 0.6, the cells were centrifuged at 4 ℃ and 4000 g for 5 min and used to prepare the MG1655-cas competent cells for electroporation.

The plasmid pTarget-X and the corresponding replacement fragment were co-transformed into MG1655-cas competent cells by electroporation and incubated at 30 ℃ and 250 rpm for 2 h and then cultured overnight on a solid LB medium containing 50 μg mL^{-1} of kanamycin and 40 μg mL^{-1} of streptomycin. The colonies in the overnight culture plates were screened by colony PCR using the corresponding sequencing primers in Table 2. Three positive clones were selected for each knockout gene and stored for cryopreservation.

1.2 Determination of cadaverine

The quantification of cadaverine was conducted by high-performance liquid chromatography (HPLC), using an Agilent (Santa Clara, CA, USA) 1290 Infinity System equipped with a fluorescence detector (FLD G1321B).

The derivatization of samples were firstly conducted. Each sample was placed in a 25 ml volumetric flask, 1 ml of 1.7 diaminoheptane (internal standard) was added, and the final volume was made up with a 0.6 N $HClO_4$ solution. An aliquot (0.5 ml) of the mixture was then immediately placed in a tube, and 100 μl of 2 N NaOH (to make the solution more alkaline), 150 μl of a saturated solution of $NaHCO_3$ and 1 ml of dansyl chloride, were added consecutively. The tube was shaken gently, and placed in a water bath at 40 ℃ for 45 min. In order to remove residues of dansyl chloride, 50 μl of ammonia were then added and the mixture was left to stand for 30 min. Finally, the volume was made up to 2.5 ml with acetonitrile and the mixture was filtered (0.25 μm).

The stationary phase was a reverse-phase column Prevail C18 (250 mm × 4.6 mm × 5 μm) (Grace, Columbia, MD, USA), and the mobile phase was a mixture of acetonitrile (A) and a solution of 0.1 M ammonium acetate (B) flowing under gradient elution. The gradient program consisted of 50% A initially, 90% A at 19 min, and 20–30 min of 50% A. An additional 5-min step was included to reach the initial conditions and achieve mobile phase stabilization. The flow rate was 1 ml min^{-1}, and the injection volume was 20 μL. The temperature of the column was 40 ± 1°C, and fluorescence was measured at an excitation wavelength of 320 nm and an emission wavelength of 523 nm.
